# Novel variants associated with premature ovarian insufficiency in Russian adolescents

**DOI:** 10.3389/fendo.2025.1687148

**Published:** 2025-11-27

**Authors:** Polina Tsabai, Zaira Kumykova, Victoria Averkova, Nadezhda Pavlova, Dmitry Maslennikov, Anna Bolshakova, Zalina Batyrova, Tamara Kolpakova, Andrey Bystritskiy, Natalia Karetnikova, Alexey Ekimov, Andrey Goltsov, Maria Kuznetsova, Anna Turchinets, Irina Mukosey, Taisiya Kochetkova, Igor Sadelov, Jekaterina Shubina, Elena Uvarova, Svetlana Yureneva, Dmitry Trofimov, Gennady Sukhikh

**Affiliations:** National Medical Research Center for Obstetrics, Gynecology and Perinatology named after Academician V.I.Kulakov of Ministry of Health of Russian Federation, Moscow, Russia

**Keywords:** premature ovarian insufficiency, ovarian dysgenesis, whole-exome sequencing (WES), *FMR1*, differences of sex development (DSD), adolescent, genetics

## Abstract

**Introduction:**

While variants in hundreds of genes have been linked to premature ovarian insufficiency (POI), monogenic disorders account for fewer than half of idiopathic POI cases in adolescents with 46,XX karyotype. This highlights the need for the further genetic investigation across diverse populations.

**Patients and methods:**

We recruited 63 Russian patients diagnosed with 46,XX POI before age 18. All underwent *FMR1* premutation testing and whole-exome sequencing (WES). Copy number variation (CNV) analysis was conducted on WES data. Segregation studies by Sanger sequencing were performed where samples from the patients’ relatives were available.

**Results:**

We identified variants in 15 genes in 38% of the cohort, including 13 causative genes (*FMR1*, *DCAF17, FOXL2, STAG3, TP63, BNC1, CPEB1, NOBOX, LMNA, FSHR, SPIDR, MCM8, EIF2B2*) and 2 candidate genes (*MYRF, LATS1*). 3.2% of patients carried an *FMR1* premutation. WES detected causative single nucleotide variants (SNVs) in 15 patients (17.5% of the cohort). CNV analysis increased the diagnostic yield to 20.6%, identifying 15q25.2 microdeletions (*BNC1*, *CPEB1*) in two patients and *FSHR* exon 2 deletion in one patient with resistant ovary syndrome. Overall, the combination of molecular genetic approaches established a diagnosis of monogenic POI (pathogenic or likely pathogenic variants) in 23.8% of adolescents with normal female karyotype. 5 patients (7.9%) carried variants of unknown significance in *FSHR*, *LMNA*, *NOBOX*, *SPIDR, LATS1* genes, warranting further investigation.

**Discussion:**

Our findings demonstrate that WES is an effective diagnostic tool for adolescents with POI and should supplement standard karyotyping and *FMR1* testing in routine clinical practice. We report several novel variants in POI-associated genes and propose new gene-disease association.

## Introduction

Premature ovarian insufficiency (POI) is a condition defined as the loss of ovarian function before the age of 40, affecting 1-3.7% of women and representing a common cause of female infertility (1). The actual definition of POI includes clinical entities named by terms ovarian/gonadal agenesis/dysgenesis, primary ovarian insufficiency, premature/primary ovarian failure, premature menopause, hypergonadotropic amenorrhea (2). POI can develop due to genetic abnormalities, autoimmune diseases, infections, or medical interventions. The mechanisms of POI include abnormal gonadal development, diminished number of primordial follicles at birth, accelerated depletion or atresia of follicles, destruction of ovarian tissue, and resistance to gonadotropins. Depending on the extent and period of the pathological process in ovaries, it manifests as primary or secondary amenorrhea (3).

In adolescents, chromosomal abnormalities (primarily Turner syndrome) account for approximately 20% of POI cases (4, 5). Non-Turner POI is rare in this age group, with an estimated prevalence of 1 in 10,000 females under 18 (6, 7). To date, variants in hundreds of genes involved in different cellular and organ processes (gonadogenesis, meiosis, germ cells differentiation, folliculogenesis and postnatal maintenance of ovaries, hormone biosynthesis, mitochondrial function, DNA damage response and repair, etc.) have been implicated in POI (4). Targeted or whole exome sequencing (WES) enables precise diagnostics of POI, prevention of comorbidities due to syndromic POI or genetic susceptibility, and prediction of residual ovarian reserve (8–11). Genetic testing has become an essential tool for identifying causative variants and providing genetic counseling for affected individuals and their families (2). Due to the advancements in diagnostics, the proportion of idiopathic POI has decreased twofold (12).

Beyong its reproductive implications, POI has detrimental effect on overall health, increasing the risk of metabolic, cardiovascular, cognitive, and psychological issues (2). It is also significantly associated with complex genetic disorders and congenital malformations (5). Furthermore, syndromic forms of POI can be inherited, potentially leading to reproductive or extragenital disorders in offspring. Since fertility is retained to some extent in POI-affected individuals, understanding the underlying genetic features is particularly important. Ovarian dysfunction may be the only symptom of multiorganic genetic disease (10). Additionally, some forms of POI (e.g. caused by variants in meiosis-related genes) are associated with negative prognosis for euploid oocyte retrieval or increased risk of miscarriage, making assisted reproductive technologies (ART) attempts with patient’s oocytes ineffective (13, 14). Therefore, molecular diagnostics can enhance genetic counselling and improve prognosis for ART in these patients (15).

Despite the abovementioned progress on genetics of POI, 36%-67% of cases remain unexplained after thorough evaluation and require further investigation (2). Several studies including patients of mixed ancestry were published and discovered novel genetic variants related to POI via WES (9, 10, 16). This highlights the need for further research on the genetics of POI in different national cohorts, especially as menstrual irregularities are common in adolescence, and observational tactics often leads to delay in POI diagnosis (17).

Here, we describe the cohort of Russian individuals diagnosed with 46,XX POI before the age of 18 and present the results of *FMR1* premutation testing and whole exome sequencing (WES).

## Patients and methods

### Ethics review committee approval

The institutional review board of the National Medical Research Centre for Obstetrics, Gynecology and Perinatology named after Academician V.I. Kulakov approved this study. This study was conducted according to the World Medical Association International Code of Medical Ethics (Declaration of Helsinki). All participants provided a written informed consent for the use of their data for scientific purposes.

### Patients

Between January 2021 and April of 2025, patients with onset of POI before 18 years old and normal female karyotype 46,XX were recruited in the study. POI was defined as primary or secondary amenorrhea/spaniomenorrhea for more than 4 months accompanied by follicle-stimulating hormone (FSH) levels ≥25 IU/l measured on at least two separate occasions, more than four weeks apart (2). Patients with known etiology of POI, such as systemic chemotherapy, radiotherapy, autoimmune disorders or extensive ovarian surgery, established chromosomal causes of POI, were excluded from the study. The following clinical data were registered for each patient: age at the time of diagnosis, type of menstrual disorder, results of hormonal studies (FSH, E2, AMH), ovarian volume and antral follicle count (AFC), anamnesis, extragenital symptoms, family history with special concern of POI, early menopause, female and male infertility, miscarriage, ethnic origin, and consanguinity. Characteristics of the cohort (age at diagnosis, levels of hormones and volumes of the right and left ovaries) are presented as median and median absolute deviation (MAD).

### Sampling and genetic testing

Samples of venous blood from the included patients were collected in anticoagulant tubes with EDTA. DNA was isolated using the PREP-MB MAX DNA Extraction Kit (DNA-Technology, Moscow, Russia) according to the manufacturer’s protocol.

### *FMR1* premutation testing

The number of CGG-repeats in the *FMR1* gene was determined by PCR amplification using specific primers, one of which was labelled with the fluorescent dye FAM. The primers targeted the promoter CGG-repeats-containing region of the gene. Following PCR, fragment analysis was conducted with DNA samples using the Nanophor 05 genetic analyzer (Syntol, Russia) to measure the length of PCR products for each allele. The number of CGG repeats in each allele was calculated based on the fragment length.

### Whole exome sequencing and chromosomal microarray analysis

WES was performed using a NovaSeq 6000 Illumina sequencer (San Diego, CA), sequencing libraries were prepared using DNA Prep (S) Tagmentation, IDT Illumina DNA/RNA UD Indexes (both Illumina), and xGen Exome Research Panel version 2 enrichment kit according to the manufacturer’s protocol. All samples were sequenced with 70×–100× coverage depth, and 10× coverage width was at least 0.95. The data were analyzed using in-house software, which included sequence alignment to the reference GRCh38 (hg38) genome, variant calling (GATK v4.5.0.0), and quality filtering. The Ensembl Variant Effect Predictor v113.3, a number of variant significance prediction algorithms (SIFT, PolyPhen-2, SpliceAI, CADD), along with OMIM, Human Gene Mutation Database (HGMD), and ClinVar were used for the annotation of variants. LOVD and other specialized databases (if present for a particular gene) were used for variant interpretation, along with the MASTERMIND genomic search engine. The genome aggregation population database (gnomAD v4.1.0) and our internal database were used to estimate the population frequencies of the identified variants. CNVs were searched using an algorithm developed by the laboratory, which is based on the application of ExomeDepth v1.1.17. The evaluation of the pathogenicity of identified CNVs is based on technical standards for interpretation and reporting of constitutional copy number variants: a joint consensus recommendation of the American College of Medical Genetics and Genomics (ACMG) and the Clinical Genome Resource (ClinGen) (18).

The clinical significance of identified variants was assessed according to the ACMG criteria (19). To prioritize potentially causative SNVs and CNVs, HPO terms (HP:0008209 Premature ovarian insufficiency, HP:0000141 Amenorrhea, HP:0000786 Primary amenorrhea, HP:0000869 Secondary amenorrhea, HP:0008232 Elevated circulating follicle stimulating hormone level) were used; variants in genes from the OMIM phenotypic series were also analyzed: PS311360, Premature ovarian failure; PS233300, Ovarian dysgenesis. For an additional search for possible causes of POI, a panel of genes from the FeRGI Database was used, specifically filtered for the POI phenotype. In the presence of additional phenotypic features (syndromic forms of POI), additional HPO terms were applied. Pathogenic, likely pathogenic variants and variants of uncertain significance (VUS) relevant to the patient’s phenotype were reported. Variants in genes of uncertain significance were considered VUS by default. The presence of clinically significant DNA copy number variations (CNVs) was confirmed by chromosomal microarray analysis (CMA; ThermoFisher CytoScan™ Optima Suite, Thermo Fisher Scientific, Waltham, MA, USA).

Validation of the variants found by WES and segregation studies in parents and siblings of the proband were performed by Sanger sequencing when possible.

## Results

### Patients’ characteristics

**Figure 1:**
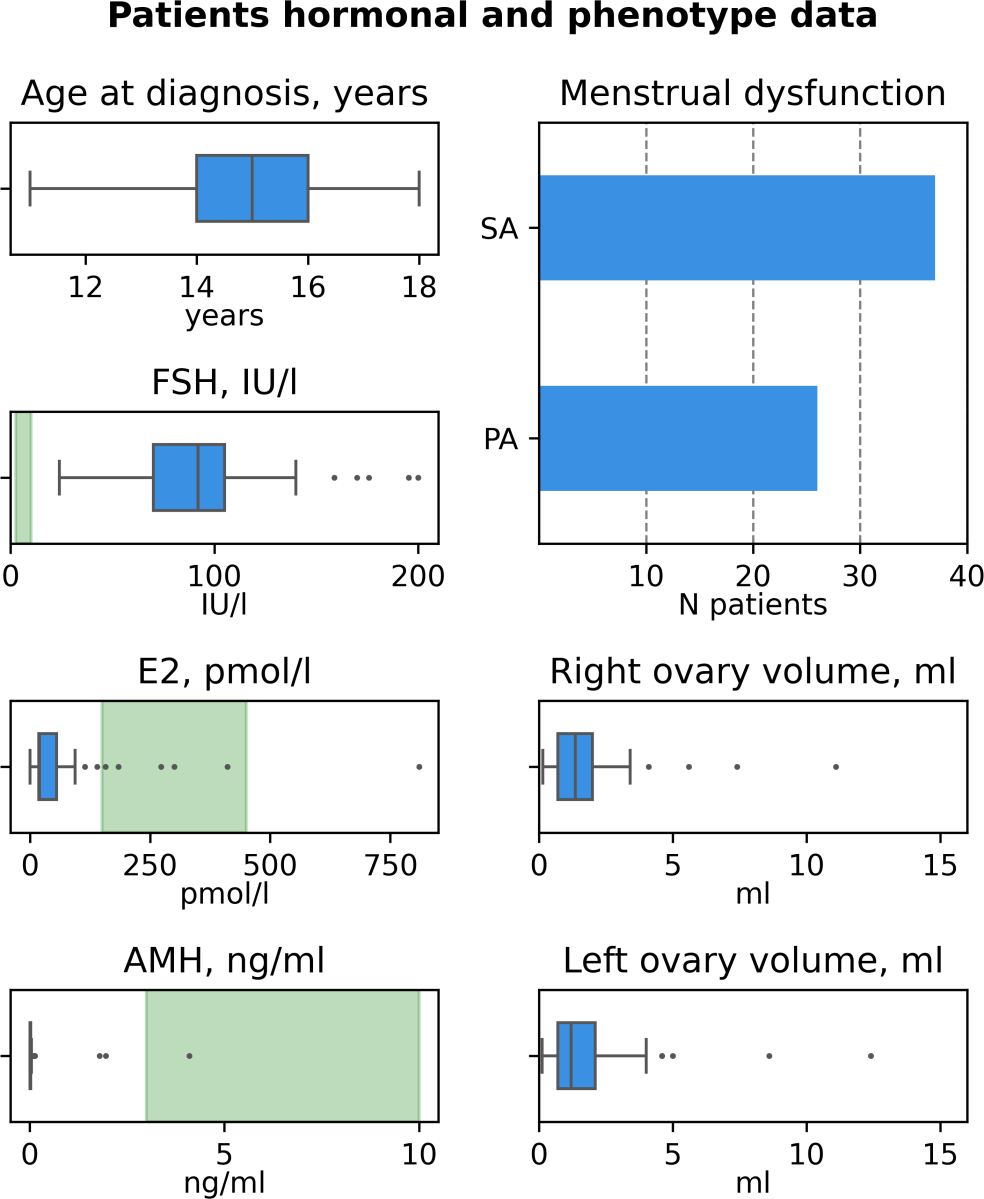
Characteristics of the cohort of patients with idiopathic adolescent-onset POI (n=63).

The characteristics of patients recruited in this study are summarized in **Table 1** and **Figure 1**, the detailed information on patients’ clinical, hormonal and anamnestic data is presented in the **Supplementary Table 1**.

**Table 1:** Characteristics of the cohort of patients with idiopathic adolescent-onset POI (n=63).

Characteristics	Median	MAD	Range
Age at diagnosis, years	15	1	11-18
FSH, IU/l	92	19	24-200
E2, pmol/l	19.81	7.79	0.1-810
AMH, ng/ml	0.01	0	0.01-4.1
Right ovary volume, ml	1.35	0.65	0.14-11.1
Left ovary volume, ml	1.2	0.6	0.12-12.4

MAD, median absolute deviation; FSH, follicle-stimulating hormone; E2, estradiol; AMH, antimullerian hormone.

The cohort comprised 63 patients from 61 families. Median age at diagnosis was 15 years old (MAD 1, range 11-18, **Figure 1A**). In 26 (41.3%) of patients POI manifested as primary amenorrhea (PA), in 37 (58.7%) – as secondary amenorrhea (SA) or spaniomenorrhea (SP) (**Figure 1B**). Median hormone concentrations were 92 IU/l for FSH (MAD 19, **Figure 1C**), 19.81 pmol/l for E2 (MAD 7.79, **Figure 1D**), 0.01 ng/ml for AMH (MAD 0, **Figure 1E**). AMH levels fell within normal range in 3 patients (P31, P49, P54). P31 and P54 also had normal AFC suggesting resistant ovaries syndrome (ROS) as a cause of POI (20). In 17 cases, ovarian tissue was visualized only on one side (27%). In 21 cases, both ovaries were not visualized (33.3%). Median volume of the right ovary was 1.35 ml (MAD 0.65, **Figure 1F**), median volume of the left ovary was 1.2 ml (MAD 0.6, **Figure 1G**). 62/63 patients (98%) were Caucasians.

**Figure 2:**
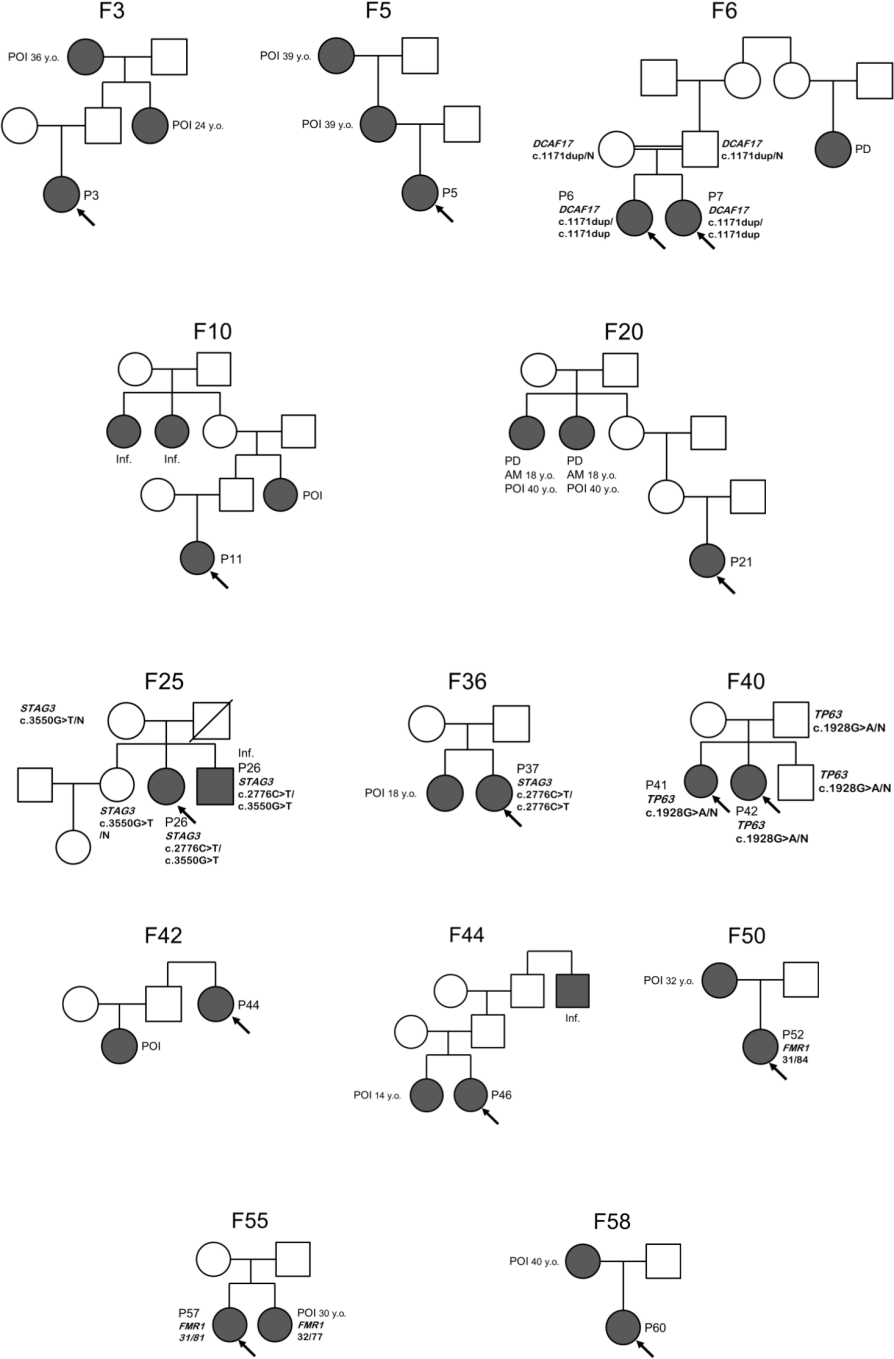
Pedigrees of familial POI cases. Proband is indicated by an arrow. Details of family history are presented in **Supplementary Table 1**. F, family; P, patient; POI, premature ovarian insufficiency; AM, age at menarche; PD, pubertal delay; Inf., infertility; N, wild type allele.

In 17/61 (27.9%) families proband’s relatives also faced reproductive issues. POI or early menopause were reported in relatives in 12 families (19.7%, for pedigrees see **Figure 2**). In 5 families, sisters were diagnosed with POI (two pairs of sisters were included in study – P6 and P7, P41 and P42). Consanguinity was reported in two families (F6 and F22), where parents were first cousins.

### Molecular genetic testing

*FMR1* alleles were normal in 60/63 (95.2%) patients. Two patients (P52 and P57, 3.2%) carried *FMR1* premutations with expansion on one allele (84 and 81 CGG repeats, respectively). The patient P14 had one allele with 45 CGG repeats belonging to the grey zone (**Supplementary Table 1**).

All patients proceeded to the WES. In total, 18 single nucleotide variants (SNV) in 12 different genes were detected in 20 patients (31.7%), 14 of which were novel variants. Causative (likely) pathogenic SNVs were found in 11/63 patients (17.5%, **Supplementary Tables 2**, **3**). 7 potentially causative variants of unknown significance (VUS) were found in 6 patients (9.5%) and corresponded to the suggested mode of inheritance. In 4 patients (6.3%) with non-syndromic POI, WES detected only single heterozygous variants in genes, associated with autosomal recessive POI (*STAG3*, *SPIDR*, *MCM8*, (21) *EIF2B2*, **Supplementary Table 4**). *FMR1* premutation testing and CNV-analysis in these cases (P9, P25, P39, P40) were negative.

Segregation studies were performed in 14 cases. Both parents were tested in 10 cases, only mother was available for testing in 4 cases. In 7 cases, the origin of the variant could not be determined, as parent(s) and siblings were unavailable for testing. Segregation analysis confirmed that the variants were in *trans* for 5 genes (*DCAF17, NOBOX, SPIDR, STAG3*). In two cases, these studies prompted the reclassification of the variants’ pathogenicity. The variant p.Ser350ProfsTer55 in the *MYRF* gene was not inherited from parents of P63, thus, appeared *de novo* and was classified as pathogenic. In patient P28, we identified the heterozygous variants in *STAG3*: VUS p.Arg360Cys and pathogenic p.Arg926Ter. According to the ACMG guidelines, variant p.Arg360Cys initially met the criteria PM2, PP2 and PP3, thus classified as a variant of uncertain significance. Sanger sequencing of family members confirmed p.Arg360Cys to be in *trans* with the previously reported variant p.Arg926Ter (PM3). Taken together, these data support reclassification of p.Arg360Cys as likely pathogenic. In family F40, the father was an asymptomatic carrier of the pathogenic heterozygous variant p.Arg643Gln in *TP63* gene. Both his daughters (patients P41 and P42) and son inherited the variants. Thus, the diagnostic value of WES after segregation analysis of SNVs was 17.5% (11/63 patients).

Furthermore, CNV analysis of WES data revealed pathogenic heterozygous microdeletions on chromosome 15q25.2, encompassing the *BNC1* and *CPEB1* genes, in two patients (P8, P10) with secondary amenorrhea. The deletions in P8 and P10 were verified with CMA (**Supplementary Table 2**). These patients had nonspecific phenotype (P8: mild eyelid ptosis, thin upper lip, smooth philtrum, streak of grey hair, P10: limited extension of elbows, hirsutism, astigmatism, tachycardia, speech delay, ADHD, pityriasis versicolor, mega cisterna magna on brain MRI). These findings increased the diagnostic performance of WES to 20.6% (13/63 patients).

Therefore, combined with *FMR1* testing results, WES established the molecular diagnosis in 15/63 patients (23.8%). Nevertheless, 39 patients (61.9%) had no candidate variants detected by WES or *FMR1* testing, including P46, who has sister with the same manifestations (ovarian dysgenesis).

It is worth noting that 9/15 (60%) patients with genetic POI suffered from autosomal recessive disorders. The consanguinity was reported only for three of them (sisters P6 and P7, P23). Among these 9 patients, there were 4 patients with *STAG3*- associated POI from unrelated families. Interestingly, all of them carried the same p.Arg926Ter variant in either homozygous or compound-heterozygous state.

The patients P6, P7, P15 and P63 had syndromic forms of POI. In these cases, clinical and molecular diagnosis was also based on specific phenotype (**Supplementary Tables 2**, **3**), and in P15 clinical diagnosis of blepharophimosis-ptosis-inverse epicanthus syndrome precluded the molecular diagnosis. According to the patient’s mother, the father had a phenotypic appearance similar to that of P15, but he was not available for examination. We assume that he may have transmitted the pathogenic variant in *FOXL2* to his daughter. Monozygotic twins P6 and P7 from a consanguineous family with a novel homozygous variant p.Glu391GlyfsTer4 in the *DCAF17* gene manifested primary amenorrhea, ovarian agenesis, hypoplastic uterus, secondary hypothyroidism, short stature, global developmental delay, mild intellectual disability, broad chest, wide-set nipples, genu valgum, broad tip of the nose, high palate, widely spaced incisors, cone-shaped fingers, consistent with diagnosis of Woodhouse-Sakati syndrome. In patient P63, a novel heterozygous variant p.Ser350ProfsTer55 in the *MYRF* gene was identified. P63 also had a congenital heart defect (secundum atrial septal defect with an absent superior rim, partial anomalous pulmonary venous return draining to the right atrium, bicuspid aortic valve) and mild hypermetropia, both of which are distinctive of *MYRF*-related disorders.

As shown in **Supplementary Tables 2** and **3**, 5 patients (7.9%) carried VUSes in known human POI-causing genes (*LMNA, NOBOX, SPIDR, FSHR)* or in genes, which disruption leads to ovarian dysgenesis in mice (*LATS1*). A heterozygous VUS p.Ala491Gly in the *LMNA* gene was identified in patient P17. The origin of the variant could not be determined, as her father died at age 52 from heart failure, and her mother was not a carrier. Further segregation studies were not possible. The homozygous in-frame deletion p.Cys310_Glu313del in the RAD51-binding domain of the *SPIDR* gene was identified via WES in patient P25. Sanger sequencing revealed that the patients’ elder fertile sister, younger brother, and both parents were heterozygous carriers of the variant. Patient P23, from a consanguineous family, harbored a homozygous missense variant p.Leu287Val in the *NOBOX* gene. The family declined further segregation studies in the patients’ elder fertile sisters. In patient P49, diagnosed with resistant ovaries syndrome (ROS) and PA, we identified a compound heterozygous SNV and CNV in the *FSHR* gene: a novel hemizygous variant p.Phe66Cys and a heterozygous deletion involving exon 2 of the gene. We could not define the precise boundaries of the intragenic deletion. P49 also carried a novel likely pathogenic heterozygous variant p.Pro2002Arg in the *FBN1* gene, which likely accounts for her ophthalmological features (OU mild myopia, OS peripheral vitreochorioretinal dystrophy).

In patient P47, we identified two heterozygous missense variants in *LATS1* gene, which is not associated with any monogenic disease, according to OMIM: c.1334C>G (p.Pro445Arg) and c.234G>T (p.Leu78Phe), which were classified as VUS. Sanger sequencing confirmed that variants are compound-heterozygous and parents are heterozygous carriers. The family history of patient P47 was unremarkable, she had no siblings. Her extragenital features included inferior vermian hypoplasia, hypermetropic astigmatism, and migraine.

Genetic POI was diagnosed more often in patients with PA (9/26, 34.6%) than with SA/SP (6/37, 16.2%). Vice versa, patients with genetic POI were more likely to manifest PA (9/15, 60%) in comparison to patients with no molecular diagnosis (17/48, 35.4%). Among 15 patients with established genetic POI, three had congenital anomalies (20%), 4 had dysmorphic features (26.7%) and 2 had developmental delay (13.3%). Among patients without established genetic cause of POI, one had congenital anomalies (2%, P59 born prematurely from complicated pregnancy had Klippel-Feil syndrome), 9 had non-specific dysmorphic features (18.7%), such as ocular hypertelorism, short palpebral fissures, high forehead, hemifacial hypoplasia, upslanting palpebral fissures, long lashes, low-set columella, smooth philtrum, thin upper lip, mandibular prognathism, short broad neck, connective tissue disorders, etc. 4 had either intellectual disability (P36) or neurological problems, e.g. migraine, diffuse muscular hypotonia, syncope (10.4%, P19, P31, P36, P54).

In 12 cases with familial POI or early menopause, the rate of genetic diagnosis (pathogenic or likely pathogenic variants) was 41.7%. This included 2 families with autosomal recessive POI (F6 with *DCAF17* variants and F36 with *STAG3* variants), one family with autosomal dominant POI (F40, paternally inherited *TP63* variant), and 2 families with *FMR1* premutation (F50, F55). Still, family history of POI or early menopause was a predictor of genetic POI diagnosis only if the proband had an affected sister [4/5 cases (80%)], but not when other relatives, e.g. mother, grandmother, aunt, were affected (1/7 cases – P52 with *FMR1* premutation has an affected mother, 14.3%). Thus, in families with intergenerational transmission, the prevalence of genetic POI was compatible to that in sporadic cases (8/49, 16.3%). Thus, in most families with intergenerational transmission of POI we did not find a molecular explanation for disorder in affected relatives.

## Discussion

In our cohort of adolescents with 46,XX POI, whole-exome sequencing yielded a diagnostic rate of 17.5%, consistent with other WES studies (9, 22, 23). Combining WES with *FMR1* testing, CNV analysis, and segregation studies established a molecular diagnosis in 15 of 63 adolescents (23.8%). Recent study by Cosette et al. using array-CGH and next-generation sequencing resulted in very similar rate of causative variants detection of 28.6%, although the average age of diagnosis was more than in our study (27.7 years vs. 15 years) (24). In our cohort 9 out of 63 cases (14.3%) were caused by variants in genes associated with autosomal recessive POI. 3 of these cases had consanguinity in family history and carried homozygous likely pathogenic variants or VUS. For *STAG3*-related POI, which showed high prevalence in our cohort, we did not find correlation with consanguinity or specific ethnic groups.

Among genetic findings, we diagnosed 3 patients with SA with fragile X-related POI (FXPOI). Early-onset secondary amenorrhea is not typical for FXPOI, and median age of amenorrhea in premutation carriers is around 38 years (25). It is estimated that approximately 3% of *FMR1* premutation carriers have irregular menstrual cycles in adolescence and only 1% of them experience final menstruation before the age of 18 (26). We performed WES in these patients, but did not find alternative monogenic cause of POI.

The etiology of POI remains unknown for 48 of 63 patients (76.2%) in whom no candidate variants were found or who carried only VUS. The possibility of other undetected variants cannot be excluded. Studies show that besides variants in single genes, many POI patients have oligogenic or multigenic contribution to phenotype (9, 27, 28). Other undetectable on WES causes may include variants in mitochondrial DNA (29), epigenetic factors (30), mosaic chromosomal aneuploidy in ovarian tissue (31). In this study, we could not establish the clinical significance of variants in *FSHR*, *LMNA*, *NOBOX*, *SPIDR, LATS1* genes, mainly due to unavailability of blood samples of proband’s relatives. Further studies and animal modelling are required to determine the real causative relationship between these variants and ovarian dysfunction that is beyond the scope of this article.

Notably, in familial cases with intergenerational inheritance of ovarian dysfunction, we identified no shared variants among affected relatives aside from the *FMR1* premutation. Rouen et al. studied 36 familial cases of non-syndromic POI and found causative variants in 50%, both with inter- and intragenerational transmission (32). Thus, within families POI and early menopause can be of different origin reflecting polyetiological nature of ovarian dysfunction.

Below we discuss the genetic variants found in our patients.

### Variants in established syndromic POI genes

Monozygotic twins P6 and P7 from a consanguineous family with a novel homozygous variant p.Glu391GlyfsTer4 in the *DCAF17* gene had a Turner syndrome-like phenotype and intellectual disability. The DCAF17 (Ddb1- and Cul4-associated factor 17) protein acts as a substrate receptor for the CUL4-DDB1 E3 ubiquitin ligase complex. The loss of its function results in Woodhouse-Sakati syndrome (WSS), a rare autosomal recessive disorder presenting with hypogonadism in both sexes, lack of secondary sex characteristics, partial alopecia, diabetes mellitus, intellectual disability, deafness, electrocardiographic abnormalities, and extrapyramidal disorders (33). Hypogonadism is a constant feature of WSS: in females, there are streak or hypoplastic ovaries with no oocytes on biopsy and hypergonadotropic hypogonadism (34, 35), while males have azoospermia and hypogonadotropic hypogonadism (36). In females, FSH can be elevated more or less significantly (37), despite there are no documented cases of normal ovarian function. All patients with WSS show ovarian dysgenesis and hypoplastic mullerian derivatives (38). Murine models support the role of this gene in ovariogenesis. Dcaf17-knockout mice are subfertile and exhibit follicular depletion at all stages (39). At presentation, our patients did not exhibit diabetes mellitus, alopecia, hearing loss, or extrapyramidal disorders. Still, different symptoms of WSS may manifest at older age: for example, neurological disorders appear in adolescence, and diabetes mellitus usually develops up to the 25 years of age (40). Thus, the results of WES suggest a risk of developing the abovementioned issues in future, so we recommended patients to assess their endocrine (screening for glucose intolerance and thyroid dysfunction), cardiological and neurological status (assessment for dystonia, dysarthria, dysphagia and hearing loss) annually.

Patient P15 presented with the facial phenotype of blepharophimosis-ptosis-inverse epicanthus syndrome and primary amenorrhea resulting from ovarian dysgenesis. WES identified the previously reported in POI heterozygous variant p.Lys193SerfsTer78 in the *FOXL2* (forkhead box L2) gene (41).

A heterozygous VUS p.Ala491Gly in the *LMNA* gene was identified in patient P17. The *LMNA* gene is associated with a wide spectrum of disorders, including premature aging syndromes. In 2003, Chen et al. described three female patients with atypical Werner syndrome and hypogonadism who carried heterozygous variants Ala57Pro and Arg133Leu in *LMNA* (42). In 2008, McPherson et al. proposed an association between a heterozygous missense variant p.Leu59Arg in *LMNA* and Malouf syndrome (dilated cardiomyopathy with hypergonadotropic hypogonadism), based on two unrelated patients with dysgenetic ovaries and onset of cardiomyopathy at ages 12 and 17 (43). In contrast, P17 showed no signs of premature aging, lipodystrophy, or cardiomyopathy. Her growth and intelligence were normal. The same variant has been reported in ClinVar in a patient with primary dilated cardiomyopathy (RCV004013487.2). Considering this, p.Ala491Gly variant was classified as VUS, and regular echocardiography monitoring was recommended for the patient. The causative relationship between *LMNA* variants and POI remains questionable, as most patients with a clinical diagnosis of Malouf syndrome have no identifiable variants in the coding regions of *LMNA* (44–46). The pathogenetic mechanism of ovarian dysgenesis in carriers of heterozygous *LMNA* variants remains unknown. Although mice lacking lamin A showed no abnormalities in ovarian morphology, spermatogenesis is severely disrupted at the pachytene stage (47). Dominant-negative variants in *LMNA* may cause Malouf syndrome through a mechanism different from the one studied by Alsheimer et al.

### Variants in established non-syndromic POI genes

The pathogenic effect of heterozygous POI-causing variants in the *TP63* gene is sex-limited. Paternal inheritance of the variant p.Arg643Gln found in patients P41 and P42 has been previously reported (48, 49), while p.Arg594Ter variant detected in P45 was described as *de novo* (50). Variants in *TP63* are associated with broad spectrum of conditions related to ectodermal dysplasia. Recently, a case series on *TP63*-associated POI showed that same variant may cause POI or isolated cleft palate in relatives, but also that non-syndromic POI may be caused by variant associated with ADULT syndrome (51), which makes the resulting phenotype prediction uncertain. Knowledge of the molecular diagnosis allows family F40 to make informed reproductive choices and to consider options such as PGT-M or prenatal diagnostics for family planning.

In contrast to the *TP63* gene, variants in genes involved in meiosis often cause infertility in both females and males (52). The *STAG3* gene encodes for the stromal antigen 3 protein, which is involved in the formation of the cohesion complex. Inactivation of Stag3 in mice results in the absence of axial elements and synaptonemal complex formation, leading to gonadal dysgenesis in both sexes (53). In the family of patient P26, who carried compound-heterozygous variants in the *STAG3* gene, segregation studies led to the identification of an affected brother with severe oligoasthenoteratozoospermia (54). The p.Arg926Ter variant in the *STAG3* gene was the most common causative variant in non-syndromic POI, found in 4 unrelated patients in our cohort, proposing p.Arg926Ter as an enriched variant in our cohort. Bergant et al. (55) have reported this variant in homozygous state in patient with PA (55). For the non-Russian population, it is the only case with this variant described to our current knowledge, which may indicate a higher prevalence in our population. However, the possibility of random fluctuation due to the limited sample size cannot be excluded, especially given that monogenic causes of POI are understudied. The variant frequency according to gnomAD v.4.0 is 0.000065, and its frequency is yet to be investigated in Russia to reveal whether or not it is a recurrent variant or just reflects chance variation due to the limited sample size. We also report a novel likely pathogenic missense variant, p.Arg360Cys, located in the armadillo domain of the *STAG3* gene, found in compound-heterozygote with p.Arg926Ter variant in patient P28. Several causative missense variants within armadillo (56–58) or STAG (57) domains of the protein have been described in publications on POI.

Similar to the variants in the *STAG3* gene, biallelic variants in the *SPIDR* gene cause both female and male infertility (59). The *SPIDR* gene encodes for a scaffold protein involved in DNA repair which is an essential factor in meiotic homologous recombination (60). The homozygous in-frame deletion p.Cys310_Glu313del in the RAD51-binding domain of SPIDR was identified via WES in patient P25. This case is of particular interest due to the rarity of *SPIDR*-associated POI: only three infertile females with biallelic *SPIDR* variants have been described up to date (61, 62). Further studies (e.g. sister chromatide exchanges) are required to determine the pathogenicity of this novel in-frame deletion.

In patients P8 and P10, CNV-analysis of WES data suggested a deletion on chromosome 15q25.2, encompassing the *BNC1* and *CPEB1* genes. Complete deletions of both genes have been reported to cause non-syndromic POI. Hyon et al. described three women with a microdeletion in the 15q25.2 region encompassing the *BNC1* and *CPEB1* genes; two of these patients exhibited primary amenorrhea (63). Similarly, in a study by Bestetti et al., microdeletions of 15q25.2 including both genes were found in two out of 67 women with POI (64). Chen et al. described a 14-year-old girl with POI and a deletion of approximately 0.447 Mb in the 15q25.2 region that, interestingly, did not include the *CPEB1* gene, emphasizing the role of *BNC1* haploinsufficiency in POI development (65). The *BNC1* (basonuclin 1) gene regulates transcription in germ cells and affects follicle development and survival. Zhang et al. reported a familial case of POI in which a heterozygous frameshift variant in *BNC1* was identified. A mouse model carrying this mutation in *Bnc1* demonstrated infertility, elevated FSH levels, decreased ovarian size, and reduced follicle count (66). Later, they showed that BNC1 deficiency triggers oocyte ferroptosis leading to premature follicular activation and excessive follicular atresia (67). The *CPEB1* gene encodes cytoplasmic polyadenylation element-binding protein 1, which regulates the polyadenylation and translation of several mRNAs important for oocyte reentry into the meiotic cell cycle. Takahashi et al. demonstrated that *Cpeb1* mRNA translation and protein levels decrease with age, resulting in altered translation in oocytes and aberrant progression through the meiotic cell cycle. They observed that *Cpeb1* haploinsufficiency caused similar changes in proteostasis in young oocytes, while increasing CPEB1 protein levels in aged oocytes rescued the translation phenotype (68). Thus, haploinsufficiency of both *BNC1* and *CPEB1* should be considered a potential mechanism for POI development; however, *BNC1* may contribute more significantly. Although the deletions found in patients P8 and P10 included other haploinsufficiency-sensitive genes, like *RPS17, HOMER2*, the patients exhibited no extragenital symptoms similar to those described in syndromic patients with 15q25.2 microdeletions (69).

In patient P49, diagnosed with resistant ovaries syndrome (ROS), we identified a compound heterozygous SNV and CNV in the *FSHR* gene: a novel hemizygous variant p.Phe66Cys and a heterozygous deletion involving exon 2 of the gene. Biallelic missense and truncating variants in *FSHR* causing POI with ROS have been reported, as well as several exon deletions in this gene (70–72). ROS usually leads to primary amenorrhea, while rare cases of secondary amenorrhea were reported (73). Interestingly, in two other patients (P31 and P54) with secondary amenorrhea clinically diagnosed with ROS, we did not identify any *FSHR* variants. Also, in P49 we found likely pathogenic heterozygous variant p.Pro2002Arg in the *FBN1* gene located outside exons 65–66 of *FBN1*, which encode asprosin, a C-terminal cleavage product of fibrillin 1 that may influence ovarian function (74), thus, it is not considered to contribute to the development of POI in this patient.

Patient P23, from a consanguineous family, harbored a homozygous missense variant p.Leu287Val located in the homeobox domain of the *NOBOX* gene. *NOBOX* (newborn ovary homeobox) is a gene involved in the earliest stages of folliculogenesis. Most reported variants are located within the homeodomain, which is responsible for nuclear localization of NOBOX protein (75). Biallelic variants in this gene have been reported as the most frequent cause of monogenic POI (76), though they were uncommon in our cohort (1/63, 1.6%).

### Potential novel gene-phenotype associations in POI

A novel heterozygous variant p.Ser350ProfsTer55 in the *MYRF* gene was identified in patient P63. MYRF (myelin regulatory factor) is a transcription factor essential for oligodendrocyte development and coelomic epithelium-derived cells proliferation and migration. Heterozygous variants in *MYRF* are associated with cardiac-urogenital syndrome, nanophthalmos and mild encephalopathy with reversible myelin vacuolization. Cardiac-urogenital syndrome is a 46,XY and 46,XX disorder of sex development caused via dysregulation of gonadogenesis and, possibly, upregulation of *CITED2* gene (77, 78). Some of the several reported 46,XX patients had ovarian and/or müllerian agenesis or hypoplasia and often were severely affected by multiple congenital anomalies leading to premature death (79, 80). Recently, Ding and Tian (81) reported a patient with hypergonadotropic hypogonadism, primary amenorrhea and mullerian aplasia. Exome sequencing revealed a *de novo* variant c.1468C>G (p.Arg490Gly) in *MYRF* gene. Importantly, this patient had no significant extragenital anomalies, except possible renal duplication. Also, we described patient who had ovarian hypoplasia with preserved ovarian function and extragenital issues, and carried a heterozygous *de novo* p.Ala440ThrfsTer2 variant in *MYRF* (82). These two cases illustrate pleiotropic effect of *MYRF* variants and high variability of associated phenotype. These cases show that precise molecular diagnosis is important in syndromic ovarian dysfunction as long as fertility management of these patients must include preimplantation genetic testing of embryos for monogenic condition (PGT-M) to prevent severe malformations in children.

In patient P47, we identified two heterozygous missense variants in *LATS1*, c.1334C>G (p.Pro445Arg) and c.234G>T (p.Leu78Phe), which were classified as VUS. *LATS1* (large tumor suppressor kinase 1) encodes for a regulator of the Hippo pathway (83) and has not previously been associated with POI in humans, while the deleterious consequences of LATS1 deficiency on ovariogenesis were demonstrated in murine models. The ablation of *LATS1* in mice causes increased germ cell apoptosis with subsequent primordial follicle loss, development of ovarian cysts and stromal tumors, and lack of mammary glands (84). The *Lats1^−/−^* mice exhibit a high perinatal mortality (85). In mice with deletions of both *Lats1* and *Lats2*, ovarian enlargement was observed along with transdifferentiation of granulosa cells into seminiferous tubule-like structures and bone tissue (86). Germline variants in the *LATS1* gene are not associated with any monogenic disease in humans (OMIM: 603473), but Kim et al. proposed that heterozygous missense variant p.Arg96Trp may be a cause of familial schwannomatosis (87). The data in the current manuscript is not sufficient for suggesting *LATS1* as a potential POI-associated gene as functional studies could not be performed, nevertheless we consider this observation to be of an interest of future studies.

## Conclusions

Our study demonstrates that whole exome sequencing combined with CNV-analysis is an effective diagnostic tool in the adolescent population with POI and should be as a supplement for standard karyotyping and *FMR1* testing. Understanding the molecular mechanisms underlying POI is essential for improving clinical management and genetic counselling, including the evaluation of reproductive risks and extragenital symptoms.

## Data Availability

The original contributions presented in the study are included in the article/**Supplementary Material**. Further inquiries can be directed to the corresponding author/s.
